# A Randomized Study of the *Glunovo* Real-time CGM Effectiveness in Individuals With Poorly Controlled Type 2 Diabetes

**DOI:** 10.1210/jendso/bvaf165

**Published:** 2026-01-12

**Authors:** Elisa Lazzaroni, Vincenzo Cimino, Alessandra Gandolfi, Camilla Tinari, Loredana Bucciarelli, Paola Morpurgo, Moufida Ben Nasr, Roberta Maria Fiorina, Ida Pastore, Laura Baruffaldi, Fabrizio Losurdo, Francesca D’Addio, Gian Vincenzo Zuccotti, Laura Montefusco, Antonio Rossi, Maria Elena Lunati, Paolo Fiorina

**Affiliations:** Division of Endocrinology, ASST Fatebenefratelli-Sacco, 20157 Milan, Italy; Department of Biomedical and Clinical Sciences, International Center for T1D, Pediatric Clinical Research Center Romeo ed Enrica Invernizzi, Università Degli Studi di Milano, 20122 Milan, Italy; Division of Endocrinology, ASST Fatebenefratelli-Sacco, 20157 Milan, Italy; Department of Biomedical and Clinical Sciences, International Center for T1D, Pediatric Clinical Research Center Romeo ed Enrica Invernizzi, Università Degli Studi di Milano, 20122 Milan, Italy; Division of Endocrinology, ASST Fatebenefratelli-Sacco, 20157 Milan, Italy; Division of Endocrinology, ASST Fatebenefratelli-Sacco, 20157 Milan, Italy; Azienda di Servizi Alla Persona, Istituti Milanesi Martinitt e Stelline e Pio Albergo Trivulzio, 20146 Milano, Italy; Division of Endocrinology, ASST Fatebenefratelli-Sacco, 20157 Milan, Italy; Department of Biomedical and Clinical Sciences, International Center for T1D, Pediatric Clinical Research Center Romeo ed Enrica Invernizzi, Università Degli Studi di Milano, 20122 Milan, Italy; Department of Endocrinology and Metabolism, Humanitas Research Hospital, 20089 Rozzano (MI), Italy; Division of Endocrinology, ASST Fatebenefratelli-Sacco, 20157 Milan, Italy; Division of Endocrinology, ASST Fatebenefratelli-Sacco, 20157 Milan, Italy; Division of Endocrinology, ASST Fatebenefratelli-Sacco, 20157 Milan, Italy; Department of Biomedical and Clinical Sciences, International Center for T1D, Pediatric Clinical Research Center Romeo ed Enrica Invernizzi, Università Degli Studi di Milano, 20122 Milan, Italy; Department of Biomedical and Clinical Sciences, International Center for T1D, Pediatric Clinical Research Center Romeo ed Enrica Invernizzi, Università Degli Studi di Milano, 20122 Milan, Italy; Department of Biomedical and Clinical Sciences, International Center for T1D, Pediatric Clinical Research Center Romeo ed Enrica Invernizzi, Università Degli Studi di Milano, 20122 Milan, Italy; Division of Endocrinology, ASST Fatebenefratelli-Sacco, 20157 Milan, Italy; Department of Biomedical and Clinical Sciences, International Center for T1D, Pediatric Clinical Research Center Romeo ed Enrica Invernizzi, Università Degli Studi di Milano, 20122 Milan, Italy; Endocrinology and Diabetology Department, Galeazzi Hospital, 20157 Milan, Italy; Division of Endocrinology, ASST Fatebenefratelli-Sacco, 20157 Milan, Italy; Division of Endocrinology, ASST Fatebenefratelli-Sacco, 20157 Milan, Italy; Department of Biomedical and Clinical Sciences, International Center for T1D, Pediatric Clinical Research Center Romeo ed Enrica Invernizzi, Università Degli Studi di Milano, 20122 Milan, Italy; Nephrology Division, Boston Children’s Hospital, Harvard Medical School, Boston, MA 02115, USA

**Keywords:** type 2 diabetes, rtCGM, Hb1Ac

## Abstract

**Objective:**

We aim to evaluate the effectiveness of the novel real-time continuous glucose monitoring (rtCGM) system “*Glunovo*” in improving glycemic control and patient outcomes in individuals with poorly controlled type 2 diabetes (T2D).

**Research Design and Methods:**

This prospective, open-label, randomized controlled trial included 172 patients with T2D from the Fatebenefratelli-Sacco Hospital in Milan. Participants were randomized into 2 groups: 86 patients received the *Glunovo* rtCGM system (case group), whereas 86 continued standard self-monitoring blood glucose with glucometers (control group). The primary outcome was the change in hemoglobin A1c levels after 6 months. Secondary outcomes included glucose metrics and patient well-being assessed by the World Health Organization-5 Well-Being Scale.

**Results:**

After 6 months, the *Glunovo* group showed a significantly higher reduction in hemoglobin A1c levels (Δ = -1.4%) compared to the control group (Δ = -0.6%). Time in range significantly increased in the rtCGM group (Δ = +18.4%). Time above range and glucose management indicator showed a greater reduction in the rtCGM group, with no changes in the time below range. Patient satisfaction increased significantly over the study period with the rtCGM system.

**Conclusion:**

The use of the *Glunovo* rtCGM system significantly improved glycemic control and patient satisfaction compared to self-monitoring blood glucose. These findings suggest that the *Glunovo* rtCGM is an effective tool for managing poorly controlled T2D.

**Clinical trial registration:**

NCT07089979

Despite recent advancements that have made glucometers smaller, faster, and more accurate, adherence to self-monitoring of blood glucose (SMBG) remains a challenge [1, 2]. The use of real-time continuous glucose monitoring (rtCGM) has been shown to improve glycemic control by reducing glycated hemoglobin (HbA1c) levels without increasing the risk of hypoglycemia in patients with type 1 diabetes (T1D) [3, 4]. However, the benefits of rtCGM extend beyond T1D and have also proven effective in patients with type 2 diabetes (T2D) treated with basal insulin. Recent studies, including systematic reviews and meta-analysis, have confirmed the potential of rtCGM in patients with T2D [5, 6]. A novel meta-analysis demonstrated that rtCGM significantly improves glycemic control in adults with T2D, although the study included patients on various treatment regimens, not exclusively those on insulin therapy [7]. Others confirmed that rtCGM is particularly effective in insulin-treated patients with T2D, leading to a significant reduction in HbA1c compared to standard SMBG [8-12]. Another crucial aspect is the improvement in patients' quality of life and psychological well-being [13]. rtCGM may reduce anxiety related to hypoglycemia and increase the sense of security and control in daily diabetes management in patients with T1D or T2D [14, 15]. Furthermore, qualitative studies have demonstrated that rtCGM enhances patients' perception of control over their condition and reduces anxiety related to glycemic fluctuations [16-18]. However, despite these benefits, the use of rtCGM in the long term presents some challenges. Some patients may find difficult to maintain long-term adherence because of discomfort with the device or for the challenge of managing continuous sensor use. Additionally, the large-scale implementation of rtCGM is influenced by economic factors and the availability of data on its cost-effectiveness [19, 20]. Considering this evidence, the present study aims to evaluate the effectiveness of the new *Glunovo* rtCGM in patients with poorly controlled T2D [21, 22]. The main objective is to explore the impact of rtCGM on improving glycemic control and behavioral habits related to self-management of the disease, as well as patients' long-term acceptance of the device [23-27].

## Materials and Methods

This study is a prospective, open-label, randomized controlled trial designed to evaluate the effectiveness of the *Glunovo* rtCGM system in patients with T2D who are not adequately controlled with their current antidiabetic therapy. The study was conducted at the Fatebenefratelli-Sacco Hospital, in Milan, Italy. over a period of 24 months following approval by the institutional ethics committee (number of ethical committee: 0023448/2024). The trial included a total of 172 patients, of whom 86 were assigned to the intervention group (cases) and monitored using the *Glunovo* rtCGM, whereas 86 patients served as controls and remained on SMBG with a standard glucometer.

### Study Design and Population

Patients were eligible for inclusion if they met the following criteria: (1) Diagnosis of T2D, (2) age greater than 18 years, (3) HbA1c between 7.5% and 11%, and (4) on basal-bolus, basal-oral, or noninsulin antidiabetic therapy. Exclusion criteria included pregnancy, HbA1c > 11% or <7.5%, and diagnosis of T1D, latent autoimmune diabetes in adults, maturity onset diabetes of the young, or other forms of hyperglycemia. Participants were randomized using a random number generator (www.randomizer.org), assigning patients with even numbers to the case group and odd numbers to the control group. Patients in the case group were provided with the *Glunovo* rtCGM system and instructed on its use, whereas control group patients continued using standard glucometers for capillary glucose measurements per their usual care. The Glunovo P3 (POCTech, China) is an rtCGM system consisting of a 14-day sensor, a reusable transmitter with up to 3 years of battery life, a dedicated smartphone application, and a web-based platform for data sharing (“Glunovo Share”). The system automatically records interstitial glucose approximately every 3 minutes (∼480 readings/day) without the need for calibration or scanning. The application provides real-time values, trend arrows, ambulatory glucose profile reports, time-in-range metrics, glycemic variability indices, and estimated HbA1c. Data can be shared with up to 10 caregivers via the GN-Carer app and with health care professionals through the Glunovo Share portal. According to manufacturer documentation, the system has a reported mean absolute relative difference of 9.89%, which is comparable to other rtCGM systems currently available. For the case group, the *Glunovo* rtCGM system was applied, and participants were trained to use the sensor, which continuously monitored their glucose levels. Patients were also equipped with a glucometer for capillary blood glucose measurements in case of hypoglycemia or hyperglycemia alerts from the sensor. Data from the rtCGM were recorded over a 180-day period, whereas the control group used standard glucometers and were instructed to perform 1 to 6 daily blood glucose measurements, depending on their therapy regimen.

### Outcomes

The primary outcome of the study was to evaluate changes in HbA1c levels after 6 months of rtCGM use compared to baseline in both the intervention and control groups. HbA1c measurements were collected as part of regular care but in our centralized hospital laboratory, which uses NGSP/IFCC-standardized HPLC methods. Assessments were performed at baseline and at the 6-month study visit, within 7 days from each visit, to ensure methodological and temporal standardization. Secondary outcomes included the percentage of time spent in the target glucose range (time in range [TIR], 70-180 mg/dL), the percentage of time spent above the target glucose range (time above range [TAR], >180 mg/dL), the percentage of time spent below the target glucose range (time below range [TBR], <70 mg/dL), mean glucose levels, glucose management indicator (GMI), sensor utilization time (% of time used), patient well-being, as measured by the World Health Organization (WHO)-5 Well-Being Scale [28] and the number of sensors used during the 6-month period. The WHO-5 well-being index was used to measure the general well-being of patients through the following questions: (1) I felt cheerful and in good spirits; (2) I felt calm and relaxed; (3) I felt active and vigorous; (4) I woke up feeling fresh and rested; (5) My daily life has been filled with things that interest me.

### Data Collection and Statistical Analysis

Patient data, including demographic information (age, sex), anthropometric measurements (weight, height), and clinical parameters (glucose levels, HbA1c), were collected from medical records. Glucose data were extracted from the *Glunovo* rtCGM system or glucometer readings, depending on the group. All statistical analyses were performed using Python (SciPy). Continuous variables were expressed as mean ± SD. To assess the normality of the data, the Shapiro-Wilk test was conducted. For normally distributed continuous variables, such as age and HbA1c, paired *t*-tests were used to compare baseline and follow-up measures within each group (cases and controls), and independent *t*-tests were performed to compare differences between the case and control groups. When data violated normality assumptions, as assessed by the Shapiro-Wilk test, nonparametric tests such as the Wilcoxon signed-rank test were used. For categorical variables, such as gender distribution, a chi-square test was employed to assess differences between the groups. If expected frequencies were too small, the Fisher exact test was used as an alternative. A *P* value < .05 was considered statistically significant. For each group, we compared baseline and follow-up measurements for primary and secondary outcomes using paired *t*-tests. The significance of differences in the changes between the groups was assessed using independent *t*-tests. Additionally, a generalized linear model was employed to adjust for potential confounders such as age, sex, and therapy changes, using HbA1c delta (difference between baseline and follow-up) as the dependent variable. The results were adjusted for covariates to evaluate the independent effect of rtCGM vs SMBG. The significance threshold was set at *P* < .05 for all analyses.

## Results

### Study Population and Baseline Clinical Characteristic

**Figure 1.**
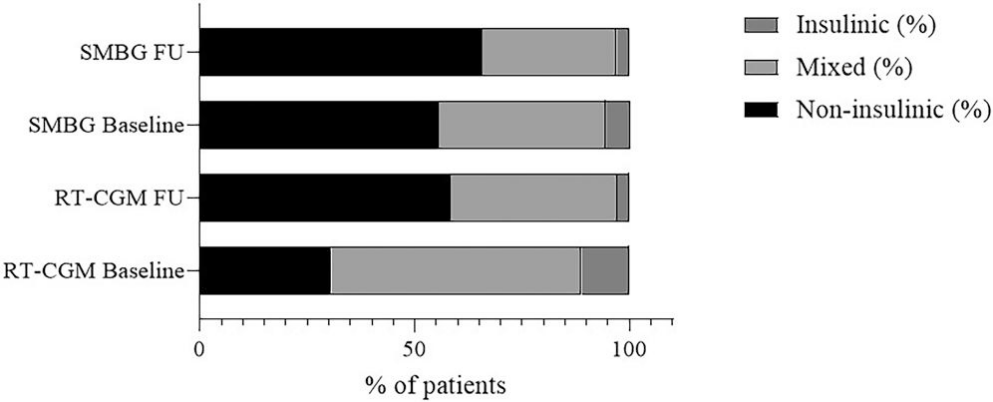
Therapy distribution at baseline and follow-up. Abbreviations: rtCGM, real-time continuous glucose monitoring; SMBG, self-monitoring of blood glucose.

The total study sample included 172 patients, randomized in a 1:1 ratio (86 per group). Seven participants in the RT-CGM arm discontinued early because of technical or organizational issues with the device, resulting in an effective follow-up population of 165 patients: 79 in the RT-CGM group and 86 in the SMBG group. The average age was 64.1 ± 8.1 years in the RT-CGM group and 69.8 ± 13.1 years in the SMBG group, with no statistically significant difference between them, with an imbalance in sex distribution. At baseline, the 2 groups were generally comparable in terms of glycemic control, being HbA1c and fasting glucose levels similar between groups. Only participants in the rtCGM group had access to CGM-derived metrics at baseline, including the GMI, TIR, TAR, TBR, and estimated glomerular filtration rate. These parameters offered a broader view of glycemic variability and renal function before the intervention. Complete baseline clinical values are reported in Table 1. Treatment regimens were categorized as insulin therapy—including basal, prandial, or basal-bolus regimens—or mixed therapy, which combined insulin with noninsulin agents (Fig. 1).

**Table 1.** Baseline and follow-up values of clinical parameters in the 2 groups (rtCGM and SMBG), with within-group comparisons

Parameter	rtCGM Baseline	rtCGM Follow-up	rtCGM *P* value	SMBG Baseline	SMBG Follow-up	SMBG *P* value
HbA1c (%)	8.1 ± 0.8	6.7 ± 0.7	*P* < .001	8.2 ± 1.0	7.6 ± 1.0	*P* < .001
Fasting glucose (mg/dL)	189 ± 43	142 ± 19	*P* = .001	180 ± 30	166 ± 32	*P* < .001
GMI (%)	7.9 ± 1.1	6.5 ± 1.1	*P* < .001	—	—	—
TIR (%)	70 ± 18	89 ± 14	*P* < .001	—	—	—
TAR (%)	25 ± 17	9.3 ± 13.3	*P* < .001	—	—	—
TBR (%)	0.4 ± 0.9	0.6 ± 2.2	ns	—	—	—
GFR (mL/min/1.73 m²)	76 ± 20	80 ± 12	ns	—	—	—

Data are expressed as mean ± SD for continuous variables.

Abbreviations: GFR, glomerular filtration rate; GMI, glucose management indicator; HbA1c, glycated hemoglobin; rtCGM, real-time continuous glucose monitoring; SMBG, self-monitoring of blood glucose; TAR, time above range; TBR, time below range; TIR, time in range.

### Follow-up Data

**Figure 2.**
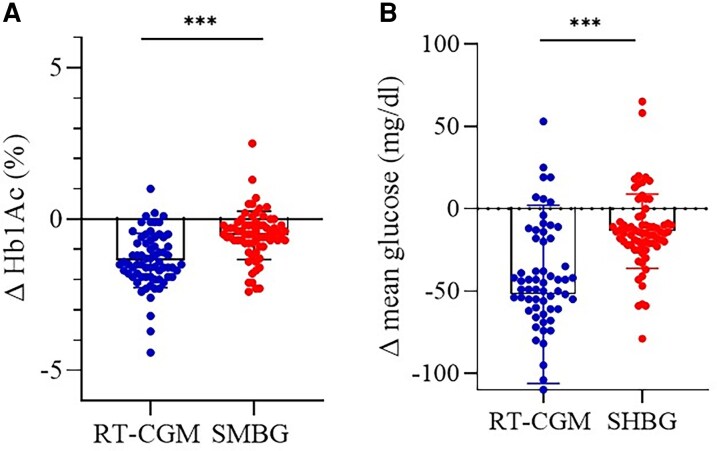
HbA1c reduction and mean glucose over time for both the case group (rtCGM) and the control group (SMBG), along with error bars representing the SDs. (A) Change in HbA1c (%). (B) Change in mean glucose (mg/dL). Data are shown as individual values with mean ± SD. ***P < .001 for between-group comparisons. Abbreviations: rtCGM, real-time continuous glucose monitoring; SMBG, self-monitoring of blood glucose.

**Figure 3.**
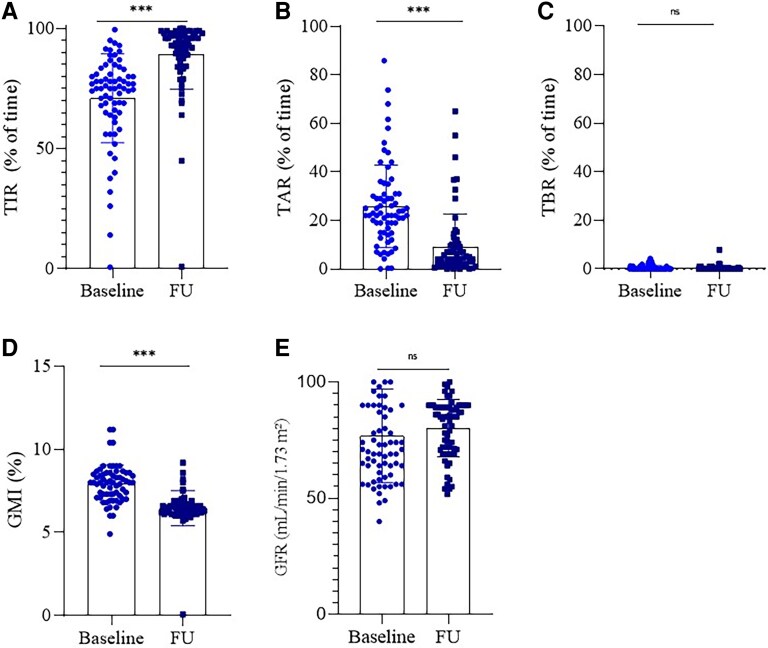
Glycemic and renal outcomes in the rtCGM group at baseline and follow-up. (A) Time in range (TIR, % of time). (B) Time above range (TAR, % of time). (C) Time below range (TBR, % of time). (D) Glucose management indicator (GMI, %). (E) Estimated glomerular filtration rate (eGFR, mL/min/1.73 m^2^). Data are expressed as mean ± SD. Statistical significance was assessed using paired *t*-tests: ***P < .001, ns = not significant. Abbreviation: rtCGM, real-time continuous glucose monitoring.

At follow-up, a significant reduction in HbA1c levels was observed in both groups. In the rtCGM group, HbA1c decreased from 8.1 ± 0.8% at baseline to 6.7 ± 0.7% at follow-up, corresponding to a reduction of ΔHbA1c = -1.4%. In the control group (SMBG), HbA1c decreased from 8.2 ± 1.0% to 7.6 ± 1.0%, with a smaller reduction of ΔHbA1c (ie, = -0.6%), as shown in Fig. 2. Patients using rtCGM also experienced substantial improvements in other glycemic parameters. Fasting glucose levels decreased significantly, GMI and TIR improved, and TAR was notably reduced, whereas TBR remained stable. Renal function, assessed via estimated glomerular filtration rate, showed a modest, nonsignificant improvement (Fig. 3). In contrast, the SMBG group showed only limited improvements, and no CGM-derived metrics were available for comparison. A complete summary of all follow-up data and statistical significance within each group is provided in Table 2.

**Table 2.** WHO-5 item scores at baseline and follow-up (rtCGM group)

WHO-5 Item	Baseline	Follow-up	*P* value
Feeling cheerful and in good spirits	3.1 ± 1.0	3.3 ± 1.2	ns
Feeling calm and relaxed	3.3 ± 1.1	3.4 ± 1.4	ns
Feeling active and full of energy	3.0 ± 1.2	3.3 ± 1.1	ns
Waking up feeling fresh and rested	3.2 ± 0.8	3.7 ± 1.0	*P* = .07
Having a life filled with things that interest the patient	3.3 ± 0.8	3.5 ± 1.0	ns

Data are expressed as mean ± SD. WHO-5 items are scored on a 0-5 Likert scale.

Abbreviations: ns, not significant; rtCGM, real-time continuous glucose monitoring; WHO, World Health Organization.

### Patient Satisfaction

**Figure 4.**
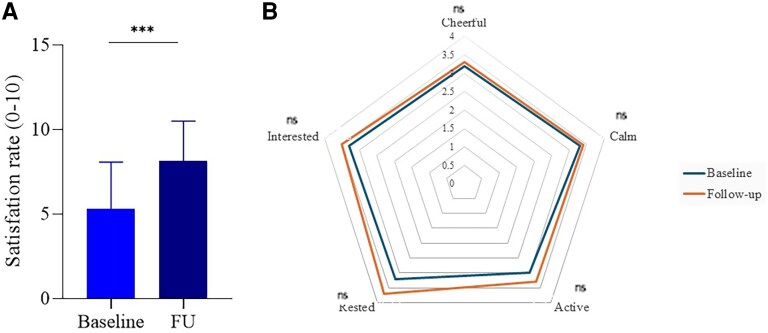
Patient satisfaction before and after rtCGM use. (A) Bar plot showing the increase in patient-reported satisfaction scores between baseline and follow-up, as measured on a 0-10 scale. (B) radar chart displaying the WHO-5 Well-Being Index for the case group at baseline and follow-up, showing the scores for each of the 5 well-being questions. Data are expressed as mean ± SD. ***P < .001; ns, not significant. Abbreviations: rtCGM, real-time continuous glucose monitoring; SMBG, self-monitoring of blood glucose.

Patient satisfaction with the sensor was assessed using 2 distinct measurement tools: a simple numeric scale (0 to 10), specifically designed to evaluate satisfaction with the sensor, and the WHO-5 Well-being Index, a validated questionnaire assessing general well-being across 5 different domains. The results showed a significant improvement in satisfaction related to the sensor. The mean satisfaction score increased from 5.2 ± 2.8 at baseline to 8.5 ± 1.9 at follow-up, indicating a substantial improvement in patients' perception of the device's utility and ease of use (Fig. 4A). However, when assessing general well-being using the WHO-5 Index, although slight improvements were observed across all 5 items after 6 months, none of the changes reached statistical significance. A numerical trend was noted particularly in the domain related to feeling rested on waking, which approached significance. Detailed item-level scores at baseline and follow-up are presented in Table 2 and in Fig. 4B.

### Multivariate Analysis of HbA1c Variation

A multivariate model was used to assess whether the observed reduction in HbA1c levels was independently associated with group allocation or influenced by confounding factors such as age, sex, renal function, or changes in therapy. The results confirmed that group allocation remained an independent predictor of HbA1c variation after adjusting for these covariates. Among the additional factors, therapy change also contributed significantly, whereas age and sex had no apparent effect. To compare the relative importance of group allocation vs therapy change, a standardized model was applied. This analysis showed that allocation to the control group had a greater influence on HbA1c variation than therapy modification. Because of the presence of outliers and deviation from normality in the residuals, a robust version of the model was also performed. Full model details are reported in Tables 3 and 4.

**Table 3.** Multivariate linear regression model for change in HbA1c

Variable	Coefficient (β)	SE	95% CI	*P* value
Control group (SMBG)	-0.717	0.184	-1.081 to -0.353	*P* < .001
Therapy change	-0.529	0.164	-0.853 to -0.205	*P* = .001
Age	Ns	—	—	ns
Sex	Ns	—	—	ns

β = linear regression coefficient; *P*-values refer to the statistical significance of each predictor.

Abbreviations: HbA1c, glycated hemoglobin; SMBG, self-monitoring of blood glucose.

**Table 4.** Standardized coefficients from the multivariate regression model

Variable	Standardized β	*P* value
Control group (SMBG)	−0.358	*P* < .001
Therapy change	−0.252	*P* = .001

Standardized coefficients (β) allow comparison of the relative strength of predictors on HbA1c variation.

Abbreviations: HbA1c, glycated hemoglobin; SMBG, self-monitoring of blood glucose.

## Discussion

This study demonstrates the significant positive impact of the novel *Glunovo* rtCGM system on metabolic control in patients with T2D. Over the 6-month intervention period, the use of the *Glunovo* rtCGM led to a pronounced reduction in HbA1c levels compared to the control group, which used standard glucometers. Specifically, the mean HbA1c reduction in the rtCGM group was -1.4%, a significant decrease that surpasses the reduction observed in the control group, where HbA1c dropped by -0.6% (Fig. 2A). These results aligned with prior findings, which reported that rtCGM use in patients with T2D resulted in more substantial reductions in HbA1c compared to those using SMBG [18]. Our data not only confirm the effectiveness of rtCGM in improving glycemic control but also suggest that this technology may induce longer-lasting behavioral changes, reinforcing better self-management practices as previously discussed [14]. The improvement in TIR observed in our study is comparable to that reported by others, who also demonstrated that rtCGM significantly enhances the time spent in the target glucose range, reducing the risk of hyperglycemia without increasing hypoglycemia [29]. The reduction in TAR and the nonsignificant change in TBR observed in our study also align with the literature. Specifically, the decrease in TAR, reinforces the effectiveness of rtCGM in preventing prolonged hyperglycemia, whereas the stability of TBR suggests that rtCGM helps to avoid the risk of hypoglycemia, a concern that has often been highlighted in previous studies [10]. The significant reduction of GMI, which provides an estimate of HbA1c levels based on average glucose readings over time among patients in the rtCGM group corroborates the HbA1c findings and suggests that rtCGM not only improves HbA1c but also helps patients maintain better long-term glucose control. Previous work showed that glycemic control can be improved when rtCGM is used in conjunction with basal insulin therapy in patients with T2D [8], with significant improvement in satisfaction scores and growing acceptance and comfort with rtCGM technology over time. Our data confirm these findings, showing that patients' satisfaction with the sensor increased, suggesting that rtCGM not only offers clinical benefits but also enhances patients' overall experience with diabetes management. To evaluate patient satisfaction, we used 2 distinct measurement tools: (1) A simple numeric scale (0-10), specifically designed to assess satisfaction with the rtCGM device; and a (2) the WHO-5 Well-Being Index, a validated questionnaire assessing general well-being across multiple psychological domains. The results showed a significant improvement in satisfaction related to the rtCGM device, as reflected by the numeric scale. However, when assessing overall well-being using the WHO-5 Well-Being Index, no significant changes were observed between baseline and follow-up. Patients reported greater satisfaction with the rtCGM device, appreciating its usability and benefits in glucose management (as measured by the numeric scale); however, this increased satisfaction with the sensor did not translate into a significant improvement in overall psychological well-being. It is possible that longer-term studies or studies using more specific psychological assessment tools could reveal different trends, as suggested in prior qualitative analyses of rtCGM's effects on patient-reported outcomes [21-23]. Future research should investigate whether extended observation periods, structured psychosocial interventions, or additional behavioral support strategies can enhance the overall well-being outcomes associated with CGM use. In conclusion, our study highlighted the clinical effectiveness of the *Glunovo* rtCGM in reducing HbA1c and increasing time in range, without increasing the risk of hypoglycemia, leading to an overall improvement in glycometabolic control. This improvement, coupled with high patient satisfaction, suggests that rtCGM can be a valuable tool in the management of poorly controlled T2D.

## Data Availability

Restrictions apply to the availability of some or all data generated or analyzed during this study to preserve patient confidentiality. The corresponding author will provide details on request regarding the conditions under which access to the data may be granted.

## Abbreviations

GMI: glucose management indicator
HbA1c: glycated hemoglobin
rtCGM: real-time continuous glucose monitoring
SMBG: self-monitoring of blood glucose
T1D: type 1 diabetes
T2D: type 2 diabetes
TAR: time above range
TBR: time below range
TIR: time in range
WHO: World Health Organization
